# Processing of complex distracting sounds in school-aged children and adults: evidence from EEG and MEG data

**DOI:** 10.3389/fpsyg.2013.00717

**Published:** 2013-10-21

**Authors:** Philipp Ruhnau, Björn Herrmann, Burkhard Maess, Jens Brauer, Angela D. Friederici, Erich Schröger

**Affiliations:** ^1^Center for Mind/Brain Science, University of TrentoMattarello, Italy; ^2^Institute of Psychology, University of LeipzigLeipzig, Germany; ^3^Max Planck Research Group “Auditory Cognition,” Max Planck Institute for Human Cognitive and Brain SciencesLeipzig, Germany; ^4^MEG and EEG: Signal Analysis and Modeling, Max Planck Institute for Human Cognitive and Brain SciencesLeipzig, Germany; ^5^Department of Neuropsychology, Max Planck Institute for Human Cognitive and Brain SciencesLeipzig, Germany

**Keywords:** auditory deviance processing, event-related potentials/fields, school-aged children, mismatch response, MMN, P3a

## Abstract

When a perceiver performs a task, rarely occurring sounds often have a distracting effect on task performance. The neural mismatch responses in event-related potentials to such distracting stimuli depend on age. Adults commonly show a negative response, whereas in children a positive as well as a negative mismatch response has been reported. Using electro- and magnetoencephalography (EEG/MEG), here we investigated the developmental changes of distraction processing in school-aged children (9–10 years) and adults. Participants took part in an auditory-visual distraction paradigm comprising a visuo-spatial primary task and task-irrelevant environmental sounds distracting from this task. Behaviorally, distractors delayed reaction times (RTs) in the primary task in both age groups, and this delay was of similar magnitude in both groups. The neurophysiological data revealed an early as well as a late mismatch response elicited by distracting stimuli in both age groups. Together with previous research, this indicates that deviance detection is accomplished in a hierarchical manner in the auditory system. Both mismatch responses were localized to auditory cortex areas. All mismatch responses were generally delayed in children, suggesting that not all neurophysiological aspects of deviance processing are mature in school-aged children. Furthermore, the P3a, reflecting involuntary attention capture, was present in both age groups in the EEG with comparable amplitudes and at similar latencies, but with a different topographical distribution. This suggests that involuntary attention shifts toward complex distractors operate comparably in school-aged children and adults, yet undergoing generator maturation.

## Introduction

Unexpected salient acoustic events can capture our attention even when they are not relevant to our current goals. This enables us to react in threatening situations, but usually at the expense of slowing down and becoming less accurate in the task pursued (Escera et al., 2000; Parmentier et al., 2008; SanMiguel et al., 2008). These effects are typically subsumed under the term distraction and are caused by the unexpected stimulus referred to as distractor.

Based on electrophysiological findings, two underlying neural processing stages of distraction have been identified (Schröger and Wolff, 1998; Escera et al., 2000; Horváth et al., 2008a). These stages are linked to temporally succeeding components in the event-related potentials (ERPs). At an early stage, deviance in the stimulation is detected and this processes is indexed by the mismatch negativity (MMN; Näätänen et al., 1978). Subsequently, attention is switched toward the distractor reflected in the P3a component (Squires et al., 1975). In the current study, we focus on these two stages and investigate the maturity of mismatch responses and P3a, and their underlying brain generators in school-aged children using magneto- and electroencephalography (MEG/EEG).

In a simple auditory-only distraction paradigm, where participants carry out a task on one auditory stimulus feature (e.g., duration), but unexpectedly become distracted by another auditory stimulus feature (e.g., pitch), deviance detection measured by the MMN is comparable in children and adults (Wetzel et al., 2006; Wetzel and Schröger, 2007a; Mikkola et al., 2010). However, more complex distractors (e.g., animal voices, tool noise, speech) presented in such a paradigm elicit a posterior deviance-related mismatch response (MMR) in children in contrast to the frontal MMN observed in adults (Gumenyuk et al., 2001; Wetzel et al., 2004; Ruhnau et al., 2010).

In a similar version of such a distraction paradigm, where participants concentrate on performing a visual instead of an auditory task, unexpected changes in the to-be-ignored auditory sequence have yielded diverging findings. For instance, a mismatch response of positive polarity (positive MMR) was observed in children younger than 10 years, whereas in adults an MMN was elicited (Ĉeponienë et al., 2004; Gumenyuk et al., 2004, 2005; Ruhnau et al., 2010). In order to explain this positive MMR in children, three competing hypotheses have been put forward. First, the temporal overlap of the component peak and a comparable maximum in the topographical distribution (disregarding the polarity) compared to the adult MMN has led to the hypothesis that the positive MMR might reflect an inverted MMN (Maurer et al., 2003). A second interpretation suggests a modulation of the sensory P2 component (Ĉeponienë et al., 2004). This hypothesis is based on comparable latencies of P2 and MMR, and similar generator structures as index by current source densities (Ruhnau et al., 2010). Finally, a third hypothesis related the MMR to an early P3a component (Ĉeponienë et al., 2004). In adults, distinct early and late P3a responses have been described (Escera et al., 1998; Yago et al., 2001), with the early P3a component showing a frontal distribution comparable to the distribution of the positive MMR in children (Ĉeponienë et al., 2004).

The current study aimed to investigate both, the positive and the negative mismatch response in the same paradigm using auditory-visual distraction (Ruhnau et al., 2010), in order to disentangle the two responses and to shed more light on the positive MMR in children.

One way to differentiate between these conflicting ERP hypotheses is to localize the underlying neural generators (Hämäläinen, 1992). The neural generators underlying the MMN have been intensively investigated in adults, localizing the main origins to supratemporal auditory cortices (for a review see Alho, 1995). Findings in children provide comparable results. In school-aged children, MMN generators have been localized to supratemporal and frontal areas (Gomot et al., 2000; Martin et al., 2003). However, the frontal source appears to develop more slowly, and thus the temporal generators are more dominant in the generation of the MMN in children than in adults. Consistent with this, the positive MMR in children has also been localized to auditory cortex areas (Gumenyuk et al., 2001; Maurer et al., 2003).

However, considering the different hypotheses concerning the underlying processes for the positive MMR (inverted MMN, P2, early P3a), source analysis using precise anatomical information in MEG (inverse) modeling is expected to provide more direct evidence compared to previous localization approaches (Gumenyuk et al., 2001; Maurer et al., 2003). Furthermore, the neural generators of the negative MMR elicited by complex sounds have not yet been localized in children.

Another ERP component commonly observed in distraction paradigms is the P3a (Escera and Corral, 2007; Wetzel and Schröger, 2007a; Horváth et al., 2008b). The P3a is elicited at around 300 ms, shows a fronto-central distribution, and has been related to an involuntary attention switch toward the distracting stimulus (Escera et al., 2000; Friedman et al., 2001; Polich, 2007). Typically, P3a amplitudes and latencies in response to a distractor are similar in children and adults indicating that mechanisms related to switching attention mature early (Horváth et al., 2009). This extends to studies presenting complex distracting sounds, which can elicit a stronger P3a at a similar latency range in children (Wetzel and Schröger, 2007b; Wetzel et al., 2011). In the current study we analyzed the P3a to replicate these previous findings, and thereby also elucidating its potential link to the positive MMR.

To sum up, the current study aimed to investigate distractor processing in children and adults, and in particular investigate whether and how the MMR dissociate (1) by measuring EEG as well as MEG, (2) by localizing the neural sources of the responses.

## Materials and methods

### Participants

Twenty adults (10 females, aged 24–34, mean 28) and 15 children (9 females, aged 9 years; 1 month to 10 years; 9 month; mean 9 years; 9 months) participated in the MEG part of the study. Ten participants from each group attended an additional EEG session at a later day (adults: 5 females, mean age 27 years; children: 4 females, mean age 10 years; 3 months). All participants were right-handed, had normal hearing and none reported a history of neurological diseases. All adult participants and, in the case of the children, their parents, gave written consent prior to testing. Adult participants were paid 7 Euro per hour, while children received a gift voucher from a local store. The study was approved by the local ethics committee of the Medical Faculty of the University of Leipzig and was conducted following the code of Ethics of the World Medical Association (Declaration of Helsinki).

### Stimuli and procedure

**Figure 1:**
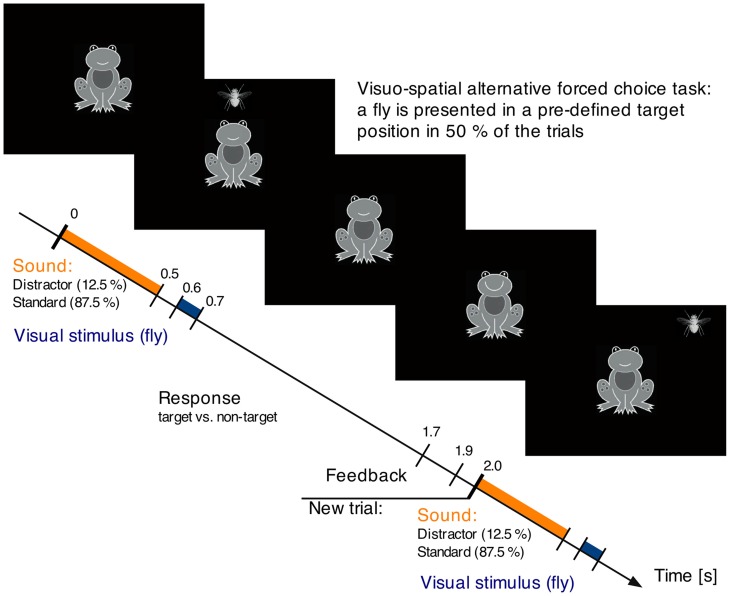
**Auditory-visual paradigm**. The participant's task is to respond to the visual stimulation and indicate whether a stimulus occurs in a target position or not. Target and non-target position appear with equal probability. Each fly is preceded by a task irrelevant sound, which is frequently a buzzing mosquito (standard), and rarely a unique environmental sound (distractor).

In both recording sessions (MEG and EEG), participants performed a visual-spatial two-alternative forced choice task (see Figure 1). A visual stimulus (a fly, 2° visual angle) was presented for 100 ms at one out of eight possible positions around a centrally presented frog. The fly could either appear at a predefined target position (*p* = 0.50), for example in the right upper corner, or at any of the other seven positions. In parallel, auditory stimuli were presented, which subjects were asked to ignore.

One trial started with an environmental sound of 0.5 s duration (10 ms rise and fall times). This sound was either a standard sound (buzzing mosquito, *p* = 0.875) or a distractor, one out of 56 uniquely appearing environmental sounds (e.g., speech, animal voices, tool noise, etc.; *p* = 0.127). All sounds were equalized for overall intensity (RMS). The distractors occurred pseudo-randomized with the first four sounds in a block being standard sounds and at least one standard was presented between two distractors. Beginning 0.1 s after the sound a fly was visually presented for 0.1 s. Participants had to indicate via button press with their index fingers whether the fly occurred in the target location or not (left/right counterbalanced across participants). Participants had 1.1 s for their response, followed by a 0.2 s visual feedback (smiling or sadly looking frog). The next trial began 0.1 s later with the presentation of the next sound. The duration of one auditory-visual trial was 2 s.

The experiment consisted of 8 blocks of ~2 min duration each, thus in total around 16 min. In each block, one spatial location on the screen served as target location. For half of the participants the order of the target locations was turning clockwise from block to block, for the other half counterclockwise. The auditory stimuli were presented 50 dB above individual hearing levels in the MEG session and at 70 dB SPL in the EEG session. The auditory stimulation was delivered via piezo loudspeakers (Model TIP-300 by Nicolet, Biomedical Madison, WI, USA) through air-conducting plastic ear tubes in the MEG session and via headphones (HD202, Sennheiser, Germany) in the EEG session. In the MEG session the visual stimuli were presented via a back projection screen (Panasonic PT-D7700E, Matsushita Electric Industrial Co., Ltd., Japan) and a mirror system while participants were in supine position. In the EEG session, visual stimuli were presented on a computer screen while participants were seated.

### MEG data recording and processing

Participants lay supine in an electromagnetically shielded room (Vacuumschmelze, Hanau, Germany). MEG signals were recorded using a Vectorview (Elekta-Neuromag Oy, Helsinki, Finland) 306-channel MEG, which comprises 204 orthogonal planar gradiometers and 102 magnetometers in 102 locations above the participant's head. The electrooculogram (EOG) was recorded by two bipolar montages, one with electrodes attached above and below the left eye, the other with two electrodes on the outer canthi.

The magnetic field recordings were sampled at 500 Hz and filtered online with a band-pass of 0.03–160 Hz. During the experiment, five head position indicator (HPI) coils measured the participant's head position continuously. After the recordings, the signal space separation method was applied to correct for head movements (using the HPIs), suppress external noise and interpolate bad channels (Taulu et al., 2004). The data was then filtered with a band-pass of 1–20 Hz (Hamming window, filter length: 879, −3 dB cutoff frequencies at 1.23 and 19.77 Hz). Epochs of 600 ms length (−100 to 500 ms time-locked to the onset of the distractor and standard sound) were extracted from the continuous data. Baseline correction was applied by subtracting the mean amplitude of the −100 to 0 ms time interval from the epoch. Epochs containing a signal range larger than 250 pT/m (gradiometer), 5 pT (magnetometer), or 150 μ V (EOG) were rejected as artifacts. Subsequently, single-trial epochs were averaged for each condition (standard, distractor). On average 245 standards (minimum 226) and 53 distractors (minimum 44) trials were included in the analysis for adults, and 186 standards (minimum 158) and of 43 distractors (minimum 35) trials for children.

### Source reconstruction, forward solution and inverse solution

Available T1-weighted MRI images of all participants, obtained with a 3-T Magnetom Trio scanner (Siemens), formed the basis for source reconstruction. The freely available software Freesurfer (http://surfer.nmr.mgh.harvard.edu) was used to construct individual cortical representations. First, non-cerebral tissue was stripped off (Ségonne et al., 2004). Secondly, the white matter was segmented and the boundary between gray and white matter was estimated. Finally, individual topological representations of the cortical surface were obtained (Dale et al., 1999). Co-registration of MRI and MEG coordinate systems was accomplished using the HPIs and about 100 digitized points on the head surface (acquired by a Polhemus FASTRAK 3D digitizer).

The MNE package provided by M. Hämäläinen (Boston, USA, http://www.nmr.mgh.harvard.edu/martinos/userInfo/data/) was used to compute forward and inverse solutions. Individual volume conductor models were created as boundary element models using the inner skull surface obtained from the T1-weighted MRI images. Using only one homogeneous volume conductor, i.e., the inner skull, has been shown to be sufficient for solving the MEG forward problem (Hämäläinen and Sarvas, 1989). As source space, the individual white matter surface was adapted by selecting about 5000 dipoles (each dipole covering an area of about 10 mm^2^) out of the ~150,000 vertices needed to describe each hemisphere.

For each participant, condition-specific inverse solutions were obtained using the standardized low-resolution brain electromagnetic tomography method (sLORETA; Pascual-Marqui, 2002). Individual cortical representations were transformed to a unique sphere representation providing a common coordinate system for all participants (Fischl et al., 1999a). The individual representations were finally morphed to the inflated cortical surface of one adult participant for purposes of visualization (Fischl et al., 1999b).

### EEG data recording and processing

The EEG recordings were acquired in an electrically shielded room. Participants were seated comfortably while electric signals were recorded from the channels C3, C4, CP5, CP6, CZ, F3, F4, F7, F8, FC3, FC4, FZ, O1, O2, P3, P4, P7, P8, PZ, T7, and T8 according to the international 10–10 system (Chatrian et al., 1988), and two additional electrodes placed at the left and right mastoid. Recordings were obtained using PORT-32/MREFA Systems (Twente Medical Systems International, Oldenzaal, Netherlands) with an input impedance of 10^12^ Ω. The EOG was recorded by two bipolar montages, one with electrodes attached above and below the left eye, the other with two electrodes on the outer canthi. The Cz electrode was used as online reference, and an electrode placed at the sternum served as common ground. Electric potentials were recorded at a 500 Hz sampling rate. EEG data analysis was carried out using the EEGLAB toolbox (Delorme and Makeig, 2004) implemented in Matlab (Mathworks Natick, MA). The EEG data were offline re-referenced to the mean of the mastoids and 1–20 Hz band-pass filtered (same settings as for the MEG recordings). Epochs of −100 to 500 ms time-locked to the stimulus onset were extracted and baseline-corrected (−100–0 ms). Epochs containing a signal range greater than 150 μV were excluded. The remaining single-trials were averaged for standards and distractors. There were on average 246 standards (minimum 233) and 53 distractors (minimum 47) trials included in the analysis for adults, and 212 standards (minimum 185) and of 50 distractors (minimum 47) remaining trials for children.

### Statistical analysis

#### Behavioral data

Reaction times (RTs) and hit rates (HRs; proportion of correct responses) from the MEG session were analyzed by means of mixed-model analyses of variance (ANOVAs) comprising the between-subject factor Age (adults; children) and the within-subject factor Stimulus Type (distractor; standard). Only correct responses were used for the RT analysis. HRs were transformed to rationalized arcsine units (rau) to account for non-normality of proportion data (Studebaker, 1985).

#### EEG amplitudes

**Figure 2:**
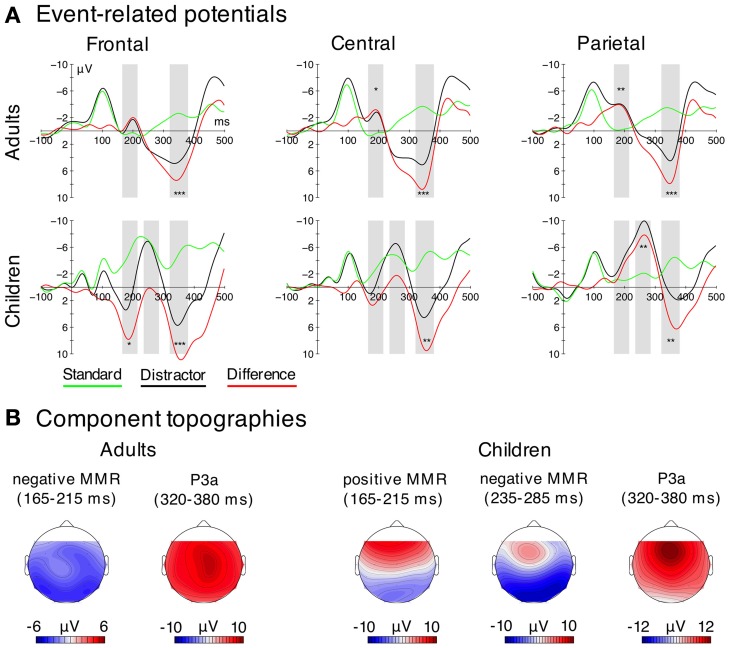
**EEG data. (A)** Grand average ERPs for both stimulus types (distractor—black; standard—green) and difference waves (distractor-minus-standard—red) for three regions used for the statistical analysis (frontal = F3, Fz, F4; central = C3, Cz, C4; parietal = P3, Pz, P4). Negativity is plotted upwards. Time windows used for the statistical analysis are highlighted in gray. Significant differences (FDR-corrected) between distractors and standards in the analysis time-windows are indicated for descriptive purposes. (^*^p_FDR_ = 0.05, ^**^p_FDR_ = 0.01, ^***^p_FDR_ = 0.001). **(B)** Electric field distributions of the negative and positive MMR and the P3a derived from the difference waves.

The effects of distractor vs. standard sounds on neural activation strength were analyzed separately for MEG and EEG. In EEG, we computed mean amplitudes over three electrode clusters (frontal: F3, Fz, F4; central: C3, Cz, C4; parietal: P3, Pz, P4) to analyze the mismatch responses and P3a amplitudes across the midline. In children, previous studies showed that the positive MMR is rather frontally distributed, while the negative MMR to complex distractors tends to be strongest at parietal electrodes (Gumenyuk et al., 2001, 2005; Wetzel et al., 2004; Ruhnau et al., 2010). Mean amplitudes were computed over different time windows centered on the peak in the grand average difference waves (cf. Figures 2, 5).

With respect to the time windows selected for the analysis, adults showed only one negative MMR (negMMR) peak, the analysis time window chosen was 165–215 ms. In children, we observed two peaks in the ERP, first a positive MMR (posMMR) followed by a negMMR. The analysis time windows were 165–215 ms (posMMR), and 235–285 ms (negMMR).

Both age groups showed a clear P3a, peaking at around 350 ms. The respective analysis time window was 320–380 ms, in agreement with previous studies (e.g., Horváth et al., 2009; Wetzel et al., 2009).

The early posMMR was analyzed in children (as it had no counterpart in adults) by means of a repeated measures (rm) ANOVA comprising the factors Stimulus Type (distractor; standard) and Electrode Cluster (frontal; central; parietal). The late negMMR and the P3a were analyzed by means of mixed-model ANOVAs comprising the between-subject factor Age (adults; children) and the within-subject factors Stimulus Type (distractor; standard) and Electrode Cluster (frontal; central; parietal).

#### MEG activation strength

In MEG, mean amplitudes derived from predefined regions of interest (ROIs) covering the peak activation from the sLORETA solutions including auditory cortex regions on the superior temporal gyrus (STG) were used for the analysis (cf. Figure 4). A ROI definition is a common step when analyzing distributed source model activity in MEG (Shtyrov et al., 2003; Palomäki et al., 2005; Orekhova et al., 2013). The selected ROIs are comparable to those chosen in previous studies investigating auditory processing (Ahveninen et al., 2011; Orekhova et al., 2013). To achieve a similar signal-to-noise ratio fed into the source analysis, signals elicited by distractor sounds were compared to signals elicited by those standard sounds that were presented directly before the distractor sounds.

Analysis time windows of the MMRs were chosen to cover the peaks in both hemispheres from the grand average difference waves in the respective age group (cf. Figure 4). In adults, this resulted in two different time windows used for analysis: one for the early MMRm (50–100 ms) and one for the late MMRm (215–265 ms). Given that the responses appeared delayed in children, the time windows chosen for the early and the late MMRm were 165–215 ms and 265–315 ms, respectively^1^. All MMRs were analyzed in mixed-model ANOVAs comprising the between-subject factor Age (adults; children) and the within-subject factors Stimulus Type (distractor; standard) and Hemisphere (left; right).

In order to link source localization to known stereotaxic coordinates, the centers of the distractor-minus-standard source activity were calculated for the early and late MMRm time windows. Mean locations of the 100 most activated vertices in the temporal cortex in the respective time windows were computed. Talairach coordinates and an approximation to Brodmann areas are provided (Talairach and Tournoux, 1988; Lancaster et al., 2000, 2007).

#### Latencies

All latencies were estimated from the distractor-minus-standard waveforms (cf. Figure 5) using a simplified jackknife approach (Smulders, 2010). The jackknife approach replaces individual average waveforms by subaverage scores computed as average across the other *n*−1 participants. The method introduced by Smulders (2010) transforms the subaverage scores to individual latency estimates, which can be submitted to statistical analyses without further adjustment.

In EEG, negMMR latencies were analyzed at the parietal electrodes (minimum in the 160–300 ms time window) and the P3a latencies were analyzed at central electrodes (maximum in the 250–450 ms time window). All latency estimates were analyzed in a One-Way ANOVA comprising the factor Age (adults; children). The posMMR was only present in children, thus no age-related latency comparison was done.

In MEG, the peak latencies of the early and the late MMRm were compared between children and adults. Early MMRm latencies were estimated as the maximum peak in the 40–200 ms time window; late MMRm latencies were estimated as the minimum peak in the 160–360 ms time window. Latency estimates were analyzed by means of a mixed-model ANOVA comprising the between-subject factor Age (adults; children) and the within-subject factor Hemisphere (left; right).

Whenever sphericity was violated (according to Mauchly's criterion) the Greenhouse-Geisser correction was applied (Greenhouse and Geisser, 1959). Corrected *p*-values and the respective epsilon coefficients are then reported. As effect size measure, the generalized eta-squared (η^2^_*G*_; Bakeman, 2005) is reported. To correct for multiple comparisons, the false discovery rate (FDR) correction was used for *post-hoc* comparisons (Benjamini and Hochberg, 1995).

## Results

### Behavioral data

The behavioral results are summarized in Table 1. The ANOVA conducted on the RTs revealed a main effect of Age Group [*F*_(1, 33)_ = 5.20, *p* = 0.029, η^2^_*G*_ = 0.129] caused by slower RTs in children compared to adults, and a main effect of Stimulus Type [*F*_(1, 33)_ = 26.10, *p* < 0.001, η^2^_*G*_ = 0.046] caused by slower RTs in trials containing a distractor than trials containing a standard. The Age × Stimulus Type interaction was not significant [*F*_(1, 33)_ = 0.05, *p* = 0.837].

**Table 1:** **Behavioral results: reaction time and hit rate results for both age groups (adults; children) and stimulus types (distractor; standard)**.

**Age group**	**Reaction times in ms**			**Hit rates in per cent**	
	**Standard**	**Distractor**	**Difference**	**Standard**	**Distractor**
Adults	430 (14)	463 (16)	33^**^	97.8 (0.3)	97.1 (0.6)
Children	487 (18)	517 (23)	30^*^	85.9 (2.1)	82.7 (2.3)

The standard error of the mean is given in parentheses. Differences (distractor-minus-standard) for reaction times including FDR corrected significance levels are presented for descriptive purposes

* *(p_FDR_ = 0.05*,

** p_FDR_ = 0.01).

The ANOVA on the rau-transformed HRs revealed a main effect of Age Group [*F*_(1, 33)_ = 61.82, *p* < 0.001, η^2^_*G*_ = 0.609], with children showing lower HRs compared to adults. Neither a main effect of Stimulus Type, nor the interaction was significant [*F*_(1, 33)_ < 2.66, *p* < 0.112].

### EEG

In the following, the results of the analyses of the event-related components are described chronologically (positive MMR, negative MMR, P3a). The ERPs are presented in Figure 2. Latency estimates are shown in Table 2.

**Table 2:** **Estimated peak latencies of analyzed components (in ms)**.

**Method**	**Component**	**Hemisphere/Electrode site**	**Adults**	**Children**	**Difference**
MEG	Early MMRm	Left	92	192	100^***^
		Right	74	–	–
	Late MMRm	Left	252	310	−58^**^
		Right	227	280	−53^***^
EEG	Positive MMR	Frontal	–	186	–
		(F3, Fz, F4)			
	Negative MMR	Parietal	199	260	−61^**^
		(P3, Pz, P4)			
	P3a	Central	346	360	14^n.s.^
		(C3, Cz, C4)			

Peak latencies of analyzed MMR components. A jackknife approach was applied to estimate the latencies (Smulders, 2010). Hemisphere is indicated for MEG, electrode cluster for EEG data. Due to the absence of the early MMRm in the right hemisphere in children and no positive MMR in adults, the respective cells where omitted from the analysis. Differences (adults-minus-children) including FDR corrected significance levels are given for descriptive purposes.

** *(p_FDR_ = 0.01*,

*** *p_FDR_ = 0.001, n.s.—not significant)*.

#### Positive mismatch response

In children, the analysis of the amplitudes in the time window of the posMMR (175–225 ms) revealed neither a main effect of Stimulus Type [*F*_(1, 9)_ = 2.66, *p* = 0.137] nor a main effect of Electrode Cluster [*F*_(2, 18)_ = 0.37, *p* = 0.624, ε = 0.700]. However, the interaction was significant [*F*_(2, 18)_ = 37.62, *p* < 0.001, ε = 0.571, η^2^_*G*_ = 0.173]. *Post-hoc* tests confirmed the positive MMR effect (larger amplitudes by distractors compared to standards) at the frontal electrode cluster [*F*_(1, 9)_ = 16.08, *p*_FDR_ < 0.050] but not at the central or parietal cluster (for both *p*_FDR_ > 0.05). These results confirm the presence of a positive MMR and furthermore its frontal distribution.

#### Negative mismatch response

The mixed model ANOVA on the amplitudes in the time window of the negMMR (adults: 175–225 ms; children: 235–285 ms) revealed a main effect of Age Group [*F*_(1, 18)_ = 13.59, *p* = 0.002, η^2^_*G*_ = 0.307], and a main effect of Stimulus Type [*F*_(1, 18)_ = 10.15, *p* = 0.005, η^2^_*G*_ = 0.087]. Furthermore, the Stimulus Type × Electrode Cluster [*F*_(2, 36)_ = 40.60, *p* < 0.001, ε = 0.690, η^2^_*G*_ = 0.061] and the Age Group × Stimulus Type × Electrode Cluster interaction were significant [*F*_(2, 36)_ = 15.25, *p* < 0.001, ε = 0.725, η^2^_*G*_ = 0.024]. To resolve the Three-Way interaction, Two-Way rmANOVAs with the factors Stimulus Type and Electrode Cluster were computed in the two age groups separately.

In adults, a main effect of Stimulus Type [*F*_(1, 9)_ = 10.65, *p* = 0.010], confirming more negative amplitudes for distractors compared to standards, and a main effect of Electrode Cluster were significant [*F*_(2, 18)_ = 9.65, *p* = 0.008, ε = 0.610]. Furthermore, the Stimulus Type × Electrode Cluster interaction was significant [*F*_(2, 18)_ = 9.34, *p* = 0.011, ε = 0.581]. *Post-hoc* tests showed larger amplitudes (i.e., more negative) for distractors compared to standards at the central [*F*_(1, 9)_ = 10.78, *p*_FDR_ < 0.050] and the parietal electrode cluster [*F*_(1, 9)_ = 39.50, *p*_FDR_ < 0.001] but not at the frontal cluster (*p*_FDR_ > 0.05).

In children, the ANOVA revealed neither a main effect of Stimulus Type [*F*_(1, 9)_ = 3.53, *p* = 0.093] nor a main effect of Electrode Cluster [*F*_(2, 18)_ = 0.62, *p* = 0.548], but a significant Stimulus Type × Electrode Cluster interaction [*F*_(2, 18)_ = 32.31, *p* < 0.001, ε = 0.650, η^2^_*G*_ = 0.177]. *Post-hoc* tests revealed that distractors elicited larger amplitudes (i.e., more negative) than standards only at the parietal cluster [*F*_(1, 9)_ = 24.65, *p*_FDR_ < 0.010], but not at frontal and central clusters (for all *p*_FDR_ > 0.05).

Taken together, these results indicate that the negMMR shows a central-parietal distribution in adults, and a parietal distribution in children (see also topographies in Figure 2).

The analysis of the peak latencies of the negMMR at the parietal electrode cluster revealed a main effect of Age Group [*F*_(1, 18)_ = 39.41, *p* < 0.001, η^2^_*G*_ = 0.687] confirming later MMR latencies in children (260 ms, SEM 3.4) compared to adults (199 ms, SEM 7.9).

#### Effects in the P3a time window

The mixed model ANOVA on the amplitudes in the time window of the P3a (320–380 ms) revealed a main effect of Stimulus Type [*F*_(1, 18)_ = 104.90, *p* < 0.001, η^2^_*G*_ = 0.559] and Electrode Cluster [*F*_(2, 36)_ = 4.49, *p* = 0.039, ε = 0.610, η^2^_*G*_ = 0.023], but no effect of age [*F*_(1, 18)_ = 0.92, *p* = 0.351]. Furthermore, the Stimulus Type × Electrode Cluster [*F*_(2, 36)_ = 11.22, *p* = 0.001, ε = 0.651, η^2^_*G*_ = 0.023] and the Age × Stimulus Type × Electrode Cluster interaction were significant [*F*_(2, 36)_ = 9.09, *p* = 0.004, ε = 0.651, η^2^_*G*_ = 0.028]. To resolve the Three-Way interaction, Two-Way rmANOVAs with the factors Stimulus Type and Electrode Cluster were carried out in the two age groups separately.

In adults, the analysis revealed a main effect of Stimulus Type [*F*_(1, 9)_ = 70.99, *p* < 0.001, η^2^_*G*_ = 0.695], confirming more positive amplitudes elicited by distractors compared to standards. Neither the main effect of Electrode Cluster nor the interaction was significant [*F*_(2, 18)_ < 1.69, *p* > 0.22].

In children, the same ANOVA revealed a main effect of Stimulus Type [*F*_(1, 9)_ = 42.88, *p* < 0.001, η^2^_*G*_ = 0.588], no main effect of Electrode Cluster [*F*_(2, 18)_ = 2.63, *p* = 0.10], but a significant Stimulus Type × Electrode Cluster interaction [*F*_(2, 18)_ = 19.65, *p* < 0.001, ε = 0.761, η^2^_*G*_ = 0.113]. *Post-hoc* tests revealed a significant P3a (larger response for distractors compared to standards) at all electrode clusters [*F*_(1, 9)_ > 25.39, *p*_FDR_ < 0.010, η^2^_*G*_ > 0.464]. However, the magnitude of the P3a effect (distractor-minus-standard) was larger at the frontal and central electrode cluster compared to the parietal cluster [*F*_(1, 9)_ > 24.03, *p*_FDR_ < 0.01], whereas the P3a effect did not differ between the frontal and central cluster (*p*_FDR_ > 0.05).

These results indicate a homogenous (central) P3a distribution in adults and a stronger fronto-centrally distributed P3a in children (see also topographies in Figure 2).

The analysis of the peak latencies of the P3a at the central electrode cluster revealed a no main effect of Age Group [*F*_(1, 18)_ = 3.03, *p* = 0.099], and thus no P3a latency difference between children and adults.

### MEG

**Figure 3:**
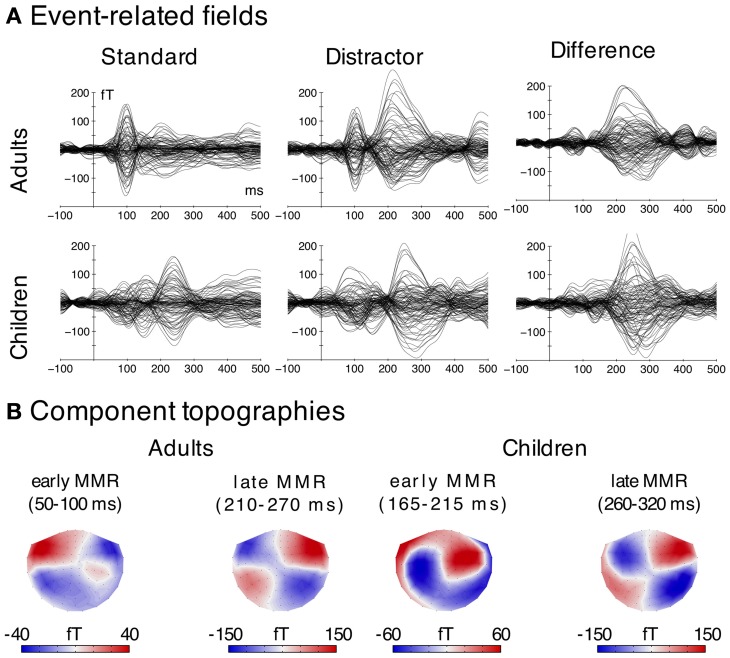
**MEG data**. **(A)** Grand average ERFs elicited by the two stimulus types (distractor; standard) and difference wave (distractor-minus-standard). Time courses for each sensor (magnetometer) are displayed. **(B)** Magnetic field distributions of the early and late MMR derived from the difference waves (distractor-minus-standard). Note that for this figure only magnetometer channels were used.

**Figure 4:**
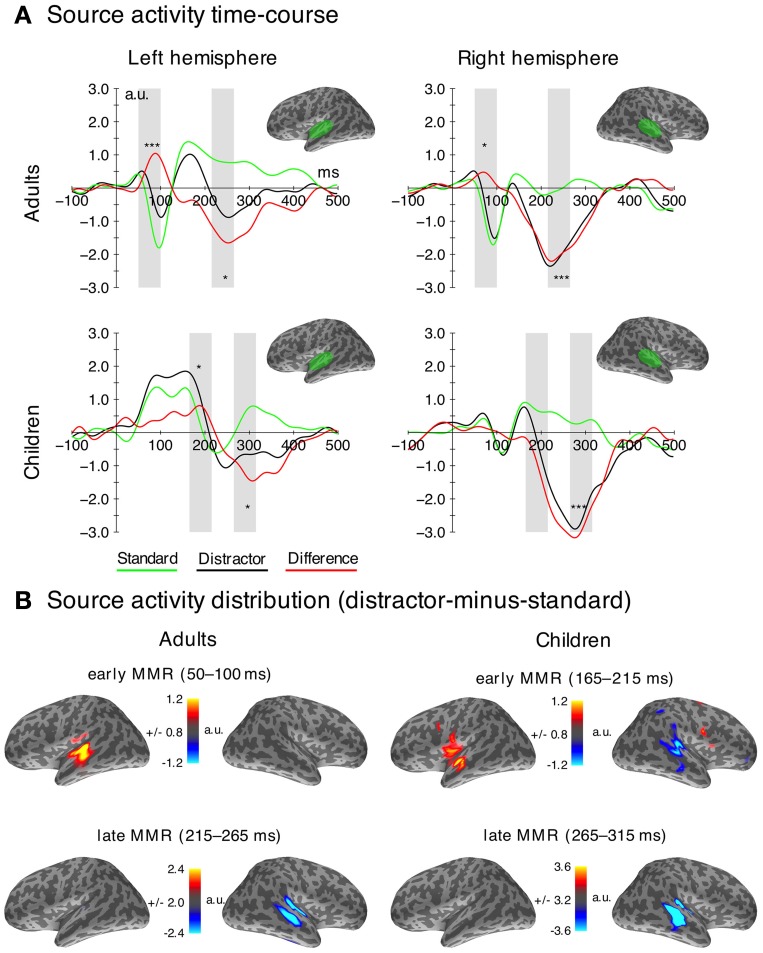
**MEG data. (A)** Time courses of the source localization for both stimulus types (distractor—black; standard—green) and difference waves (distractor-minus-standard—red) in the ROIs (marked green on top) used for the statistical analysis. Time windows used for the statistical analysis are highlighted in gray. Significant differences (FDR-corrected) between distractors and standards in the analysis time-windows are indicated for descriptive purposes. (^*^p_FDR_ = 0.05, ^***^p_FDR_ = 0.001). **(B)** Grand average source localization (distractor-minus-standard) of the early and late MMR components (a.u.—arbitrary units).

In the following sections, the results of the analyses of the event-related fields are reported chronologically (early and late MMR). ERFs are presented in Figure 3, the corresponding source activation and distribution is presented in Figure 4. The mean of the source activation for each component is presented in Table 3. Latency estimates are presented in Table 2.

**Table 3:** **Mismatch response—center of mass activity for sLORETA solutions**.

**Age group**	**Component (time window)**	**Talairach coordinates (mm)**			**Cortical region**
		***X***	***Y***	***Z***	
Adults	Early MMRm	−52	−24	5	Left BA 41 (PAC)
	(50–100 ms)	55	−16	4	Right BA 22 (STG)
	Late MMRm	−45	−26	4	Left BA 41 (PAC)
	(200–270 ms)	42	−25	5	Right BA 22 (STG)
Children	Early MMRm	−54	−9	−3	Left BA 22 (STG)
	(170–210 ms)	52	−30	9	Right BA 41 (PAC)
	Late MMRm	−43	−31	9	Left BA 41 (PAC)
	(260–320 ms)	46	−29	3	Right BA 41 (PAC)

Mean of mass activity for the sLORETA solutions. The coordinates reflect the mean of the 100 most activated vertices (absolute values) of the grand average source localization in the distractor-minus-standard differences (BA, Brodmann area; PAC, primary auditory cortex; STG, superior temporal gyrus).

#### Early mismatch responses in adults and children

The early MMR was observed in both age groups. In adults, it was generated in an area covering the superior temporal gyrus and sulcus (STG/STS) including the primary auditory cortex (PAC). In children, the response was only observed in the STG/STS in the left hemisphere and at a later latency range.

Formally, the mixed model ANOVA on the amplitudes in the early MMR time window (adults: 50–100 ms; children 165–215 ms) revealed a main effect of Age Group [*F*_(1, 33)_ = 15.22, *p* < 0.001, η^2^_*G*_ = 0.149]. Neither the Stimulus Type, nor the hemisphere effect was significant [*F*_(1, 33)_ < 3.7, *p* > 0.07]. Furthermore, the Age Group × Stimulus Type [*F*_(1, 33)_ = 8.17, *p* = 0.007, η^2^_*G*_ = 0.016], the Stimulus Type × Hemisphere [*F*_(1, 33)_ = 12.07, *p* = 0.002, η^2^_*G*_ = 0.026] and the Age Group × Stimulus Type × Hemisphere interaction were significant [*F*_(1, 33)_ = 4.46, *p* = 0.042, η^2^_*G*_ = 0.011]. To resolve the Three-Way interaction, two rmANOVAs were computed for the adults and children separately.

In adults, the analysis of the amplitudes in the early MMRm time window revealed a main effect of Stimulus Type [*F*_(1, 19)_ = 39.78, *p* < 0.001], a main effect of Hemisphere [*F*_(1, 19)_ = 5.44, *p* < 0.031] and a Stimulus Type × Hemisphere interaction [*F*_(1, 19)_ = 5.93, *p* = 0.025]. *Post-hoc* tests showed larger amplitudes for distractors compared to standards in both hemispheres [*F*_(1, 19)_ = 37.86, *p*_FDR_ < 0.001; *F*_(1, 19)_ = 10.43, *p*_FDR_ < 0.050], but this effect (distractor-minus-standard) was larger in the left compared to the right hemisphere [*F*_(1, 19)_ = 5.93, *p*_FDR_ < 0.05].

In children, neither the Stimulus Type, nor the Hemisphere main effect were significant [*F*_(1, 14)_ < 0.27, *p* > 0.614]. However, the Stimulus Type × Hemisphere interaction was significant [*F*_(1, 14)_ = 6.61, *p* = 0.022, η^2^_*G*_ = 0.032]. *Post-hoc* tests revealed larger amplitudes elicited by distractors compared to standards in the left hemisphere [*F*_(1, 14)_ = 6.75, *p*_FDR_ < 0.05], but no differences in the right hemisphere (*p*_FDR_ > 0.05).

These results indicate that the early adult MMRm is present in both hemispheres, although slightly stronger in the left, whereas the early MMRm in children is confined to the left hemisphere.

The early MMR showed clear peaks in both hemispheres in adults, but only one peak in the left hemisphere in children (cf. Figure 4). As a result, the jackknife procedure to estimate latencies failed in the right hemisphere in children. Hence, only one ANOVA for the Age Group analysis was carried out in the left hemisphere. A separate ANOVA to test Hemisphere effects was computed in adults only. The ANOVA on the peak latency of the early MMRm effect revealed a significant Age Group effect [*F*_(1, 33)_ = 159.27, *p* < 0.001, η^2^_*G*_ = 0.824], indicating much later peak latencies in children compared to adults (cf. Table 2). The ANOVA on the early MMR peak latencies in adults revealed a significant Hemisphere main effect [*F*_(1, 19)_ = 14.05, *p* < 0.010, η^2^_*G*_ = 0.209] caused by later peak latencies in the left compared to the right hemisphere. See also Table 2.

#### Late mismatch response

The late MMRm in both age groups was mainly generated in auditory areas on the STG/STS including PAC. The activation appeared stronger in the right hemisphere in both age groups (Figure 4). Our results show an age related delay of the late MMR, while the magnitude and source distribution seem to be similar.

Formally, the mixed-model ANOVA on the late MMRm (adults: 215–265 ms; children: 265–325 ms) revealed no main effect of Age Group [*F*_(1, 33)_ = 0.22, *p* = 0.642], but a main effect of Stimulus Type [*F*_(1, 33)_ = 42.46, *p* < 0.001, η^2^_*G*_ = 0.317] and a main effect of Hemisphere [*F*_(1, 33)_ = 20.02, *p* < 0.001, η^2^_*G*_ = 0.131]. Only the Stimulus Type × Hemisphere interaction was significant [*F*_(1, 33)_ = 13.82, *p* = 0.001, η^2^_*G*_ = 0.034]. *Post-hoc* comparisons revealed significant MMRs (distractors more negative than standards) in both hemispheres [left: *F*_(1, 34)_ = 16.89, *p*_FDR_ < 0.010 right: *F*_(1, 34)_ = 61.38, *p*_FDR_ < 0.001], however, the MMR effect (distractor-minus-standard) was larger in the right compared to the left hemisphere [*F*_(1, 34)_ = 12.79, *p*_FDR_ < 0.010].

The ANOVA on the peak latency of the late MMRm revealed a significant Age Group effect [*F*_(1, 33)_ = 24.90, *p* < 0.001, η^2^_*G*_ = 0.326], caused by later peak latencies in children (mean 295 ms) compared to adults (mean 240 ms). Furthermore, a significant Hemisphere effect was observed [*F*_(1, 33)_ = 16.87, *p* = 0.014, η^2^_*G*_ = 0.110] due to earlier peak latencies in the right (251 ms) compared to the left hemisphere (281 ms). The interaction was not significant [*F*_(1, 33)_ = 0.01, *p* = 0.919]. See also Table 2.

## Discussion

The current study investigated distraction processing of complex environmental sounds in school-aged children and adults. The results can be summarized as follows: (1) Behaviorally, the effects of distraction were comparable in the two age groups, yet children responded slower and less accurately in general. (2) Electrophysiological results indicate early deviance processing in adults at around 70 ms. (3) In children early deviance processing was evident at around 190 ms. (4) Both age groups showed a late effect of distractor processing, with comparable amplitudes and neural sources, however, delayed latencies in children. In the following, we discuss the results in more detail.

### Behavioral effects of distraction

The accuracy data revealed that children in general performed at a lower level compared to adults. However, no effect of the task irrelevant sounds on the primary task performance was observed, i.e., performance did not differ in trials containing distractors compared to trials containing standards.

On the other hand, reaction time data revealed that distractors impaired performance in the visual primary task. RTs were delayed in trials containing a distractor as compared to trials containing a standard sound. This is well in line with previous findings in adults (Schröger and Wolff, 1998; Escera et al., 2000; Parmentier et al., 2008; SanMiguel et al., 2008) and children (Gumenyuk et al., 2004, 2005; Wetzel and Schröger, 2007b; Wetzel et al., 2009), and confirms the distracting influence of task-irrelevant complex environmental sounds. Interestingly, the reaction time distraction effect (distractor-minus-standard) was similar between adults and children (around 30 ms), although children were generally responding slower to the visual targets by about 50 ms. In line with the current findings, reaction time distraction effects caused by complex sounds were previously reported to be of similar magnitude in adults and children (Gumenyuk et al., 2004; Wetzel et al., 2009). Thus, from behavioral findings it appears that distraction by complex sounds is mature by the age of 9–10 years. Our electrophysiological data, on the other hand, provide more detailed insight (see below).

### EEG indices of distractor processing

In what follows, we discuss the results of the different ERP effects chronologically, starting with the positive and negative MMR (early and late, respectively) followed by the P3a.

#### Positive mismatch response in children

We observed a positive, frontally distributed MMR in children but not in adults (here in the 175–225 ms time window, reliably elicited in 9 out of 10 children). This posMMR has repeatedly been reported in the literature (Maurer et al., 2003; Ĉeponienë et al., 2004; Gumenyuk et al., 2004; Wetzel et al., 2009; Ruhnau et al., 2010), and as one hypothesis, has been interpreted as an inverted MMN (Maurer et al., 2003). The cause for such a complete polarity inversion, however, has not been strongly discussed. Progression of cortical folding could explain changes in topographical distributions (Moore and Guan, 2001), but it seems unlikely that cortical areas entirely change orientation from late school age to adulthood.

Another proposal suggests that the positive MMR reflects an early P3a, a mechanism governing or initiating an attention shift to the task irrelevant deviant (Ĉeponienë et al., 2004). Yet, it seems unlikely that attention can be shifted before the deviance is detected as reflected in the subsequent negative mismatch response.

Given the close temporal proximity (or overlap) of the positive MMR and the P2, a more likely interpretation is that the positive mismatch response reflects or relates to a modulation of the P2 component (Ĉeponienë et al., 2004; Ruhnau et al., 2010). Results from current source density maps (Ruhnau et al., 2010) and the generator structure of the P2 (Crowley and Colrain, 2004) support this explanation, and the current study provides additional localization support consistent with P2-related activity (see below).

#### Negative mismatch response in children and adults

A negative posteriorly distributed MMR was observed in adults as well as in children. This effect has previously been related to deviance detection and the mismatch negativity (MMN) as the classical deviance response (Maurer et al., 2003; Wetzel et al., 2004). The distribution, however, is rather atypical for the adult MMN to pure tones (usually fronto-central maxima, e.g., Alain et al., 1998; Escera et al., 2000). Nevertheless, for complex sounds parietally distributed deviance-related effects have been reported previously, such as to personal significant deviant ring tones (Roye et al., 2007), lexical deviants (Muller-Gass et al., 2007b), and familiar environmental sounds (Kirmse et al., 2009, 2012). Moreover, the discriminative or target-specific N2 sometimes has a central-posterior maximum (Folstein and Petten, 2008). It is therefore possible that deviance-related effects reveal a more posterior distribution with spectrally complex than with spectrally simple sounds. Alternatively, it could be that with this sort of motivationally relevant sounds discriminative processes are triggered even when no task is related to the deviance, which resemble the ones that are triggered when the deviance is task-relevant. Such sounds may bear processing affordances on their own. Thus, the present effect could be a combination of MMN and N2, in both children and adults. The even more posterior distribution in children could either be a result of the overlap with the posMMR and the P3a, affecting the MMR mostly at frontal sites, or reflect the fact that children process such sounds more target-like than adults (see Figure 2).

The auditory N2 is a marker of a discriminative process (similar to the MMN, Ritter et al., 1984) and/or the registration of task relevant events (for a review see Folstein and Petten, 2008). Although distractor sounds in the current paradigm were not task relevant, their salience makes them relevant to the second and automatic task of novelty detection in the auditory modality. Taken together, the current results suggest that the posteriorly distributed negMMR is not specific to children and likely represents a combination of MMN and N2.

While we did not observe any age-related amplitude differences between age groups, the response latency of the negative MMR was delayed by about 60 ms in children. Latencies of evoked responses are affected by myelination, such that progression of myelination over childhood results in shorter ERP latencies (e.g., Gilley et al., 2005; Moore and Linthicum, 2007; Mahajan and McArthur, 2012). The latency delay of the MMR may thus indicate that myelination in generator structures underlying the MMR is not yet adult-like by the age of 9–10 years. This would be in line with studies showing that the auditory cortex is developing slowly—as opposed to other sensory cortices—well into later childhood (Moore and Linthicum, 2007). On the other hand, the partial temporal overlap of posMMR and negMMR might affect the latency of the latter similarly to maturational changes of auditory P1 superimposed by the N1 (Ponton et al., 2002). In our case, the magnitude and latency changes of the posMMR from childhood to adulthood are superimposed on the negMMR, which might add to the age related latency delay.

#### Attention shift reflected in P3a responses

The P3a was observed in both age groups at comparable latencies, similar to the P3a latencies previously reported (Horváth et al., 2009; Wetzel et al., 2009). The P3a is implicated in attention shift processes, and the current results thus indicate that the mechanism of orienting attention to irrelevant stimuli is mature in by the age of 9–10. Interestingly, the P3a latency was similar between children and adults although the preceding negMMR is clearly delayed in children. This further corroborates findings indicating that P3a and negMMR reflect independent processes (Muller-Gass et al., 2007a; Horváth et al., 2008b), which show different developmental time courses. It is noteworthy that, in contrast to the negative MMR, RT distraction effects seem to behave comparable to P3a latencies. It has been show previously that RTs and P3a are modulated by experimental manipulations, such as working memory load, in distraction paradigms while the MMN was not (e.g., Berti and Schröger, 2003; SanMiguel et al., 2008). Our results are in line with these data indicating a coupling of behavioral distraction and attentional orienting as indexed by the P3a but not to the deviance detection/classification mechanism indexed by the MMR.

Although of similar latency, the topography of the P3a was different for children and adults with children showing a more frontally distributed P3a as compared to adults (cf. Figure 2). This might be partly due to the overlap of the central-parietal negMMR with the P3a. Topographic differences of the P3a between children and adults have been reported recently (Wetzel et al., 2009), but the more frontally distributed P3a in children in the current study could also be in part due to the spatio-temporal overlap with the central-parietal negMMR. Thus, it remains for future studies to investigate whether the generator structure underlying the P3a is fully developed in middle school age.

### MEG indices of distractor processing

The MEG data revealed two different early MMRms in children and adults and one late MMRm in both age groups and no indicator of the P3a. In the following we discuss the components in chronological order.

#### Early magnetic mismatch responses

We observed a very early MMRm in adults at a peak latency of around 65 ms. In line with previous studies, this finding indicates very early deviance-related responses (Boutros and Belger, 1999; Herrmann et al., 2011; Grimm et al., 2011). Importantly, in order to control for potential effects of frequency-specific neural adaptation underlying deviance-related responses (May and Tiitinen, 2010; Herrmann et al., 2013), the current study used a broadband standard sound (buzzing mosquito) that uniformly adapts tonotopically-organized regions of auditory cortex (see e.g., Wetzel et al., 2009; Ruhnau et al., 2010), and thus reduces the likelihood of response adaptation effects. Yet, slight variations in the spectral power of the standard vs. distractor sounds require further research on the contribution of frequency-specific adaptation to the current deviance response.

**Figure 5:**
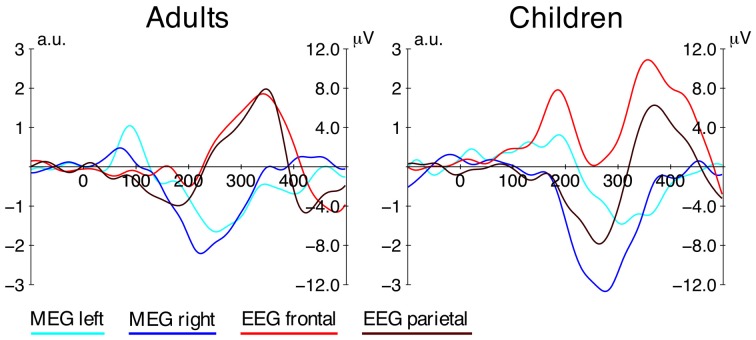
**Grand average difference waves from MEG and EEG**. Difference waves for both age groups (distractor-minus-standard) from the MEG source localization data (left and right ROI) and frontal (F3, Fz, F4) and parietal (P3, Pz, P4) electrode clusters from the EEG data. The left axis shows the amplitude for the MEG data, the right axis shows the amplitude for the EEG data (note that the y-axis is not inverted as opposed to other graphs presented here).

In contrast to the very early effect observed in adults, the earliest statistically significant effect in children occurred at around 190 ms, thus in a comparable time window as the posMMR in the EEG data^2^ (cf. Figure 5). The source distribution revealed activation in the left STG/STS and it appears that more anterior parts of the STG/STS were activated, compared to other components such as the N1 (Hari et al., 1987; Pantev et al., 1991). This observation would be in line with the hypothesis that P2 responses underlie the early mismatch effect in children (Ĉeponienë et al., 2004; Ruhnau et al., 2010; see also discussion above) because previous research localized the generator of the P2 in adults in more anterior parts of the STG as well (Hari et al., 1987; Pantev et al., 1991). On the other hand, the source localization of the early MMR and late MMR show overlapping activation in STG/STS, which is in line with previous findings of superior temporal plane activations underlying the MMN in children (Gomot et al., 2000; Martin et al., 2003). Based on these localization results, the early MMR might well-reflect a deviance detection mechanism similar to the MMN followed by the late MMR (see below).

#### Late magnetic mismatch response

A late MMRm of comparable magnitude, source location (STG/STS), and right-hemispheric predominance was observed in both age groups. As in the EEG data, the peak latencies, however, were clearly delayed in children (children: 295 ms, adults: 240 ms; this age-related latency difference was very consistent across subjects in EEG and MEG). Increased neural activation in this time window elicited by distractors has been linked to deviance detection as well as target discrimination (Ritter et al., 1984; Folstein and Petten, 2008). Thus, while deviance-related processes activate the same regions in children and adults, only the processing speed seems to be immature in children.

## General discussion: bringing EEG and MEG together

In the current study, both neurophysiological measures, that is EEG and MEG, indicate that deviance detection is still immature in school-aged children.

MEG data revealed an early MMRm (before 100 ms) in adults, which is indicating a very early deviance detection mechanism (Grimm et al., 2011). The magnetometer topographies (Figure 3) and the modeled superior-oriented source activation would suggest a positive component in EEG, which, however, was not observed in that time window. This indicates higher sensitivity of MEG to this effect, which could be due to the fact that MEG intrinsically suppresses deep and radially oriented brain activity whereas EEG is influenced by this activity, which in turn results in a lesser SNR for the EEG (Ahlfors et al., 2010a,b).

Children also showed an early MMRm (190 ms), which is, however, clearly dissociated from the very early MMRm in adults by its latency. The latency, magnetometer topographies (Figure 3), and the superior-oriented source activation indicate that this MMRm in children is corresponding to the posMMR in EEG. This component seems to be generated mainly in the left STG/STS, which is in line with research on the cortical generators of the P2 in adults (Hari et al., 1987; Pantev et al., 1991). It has been proposed that the access to the sensory representation of a complex distractor differs from that of a frequent standard reflected in a P2 modulation (Ruhnau et al., 2010). In adults, no effect of similar source activation/polarity was observed in that time window. This indicates that either this step is integrated in another process (e.g., MMN/N2) or it is diminished until adulthood. Future studies on older children are required to identify the developmental time-course of this component.

The late MMRm observed in MEG seems to have its counterpart in the electric negative MMR (see Figure 5). The underlying function of this MMR has been linked to deviance detection and stimulus/target selection mechanism attributed to the MMN/N2 (Ritter et al., 1984; Folstein and Petten, 2008). Source reconstruction based on the MEG data revealed that this response is generated in auditory areas in both age groups. It is noteworthy that EEG and MEG capture a rather identical age-related delay of the MMR (≈60 ms). This is a clear indicator that the deviances detection mechanism is not mature by the age of 9–10 years, and that neural efficiency is well-improving into later childhood.

Interestingly, the involuntary attention shift as indicated by the P3a is accomplished at a comparable latency range in children and adults. Considering that the late MMR, reflecting deviance detection, is clearly delayed in children it could be argued that children shift their attention faster than adults to yield similar P3a latencies. Yet, MMR and P3a are not necessarily reflecting serial processes, but instead partially independent ones (Horváth et al., 2008b). Furthermore, earlier processes of deviance processing are present in children as reflected by the early MMR. This indicates that deviance detection has been accomplished to a certain extent prior to the involuntary attention shift.

## Conclusion

Using EEG and MEG, the current study investigated deviance processing in school-aged children and adults. Our results show that a positive (early) as well as a negative (late) mismatch response is elicited by distracting stimuli. Hence, the current findings provide evidence for a temporal dissociation of deviance processing; both mismatch responses localized to auditory cortex areas. Interestingly, these effects were not only observed in children, but were found in MEG data of adults as well. This indicates that the positive mismatch response is not children-specific. Yet, the effects were in general delayed in children, suggesting that not all neurophysiological aspects of deviance detection are matured in school-aged children. Furthermore, the generators underlying the neural responses related to involuntary attention shifts (P3a) undergo changes in from childhood to adulthood, while response magnitude and latency are comparable in children and adults.

### Conflict of interest statement

The authors declare that the research was conducted in the absence of any commercial or financial relationships that could be construed as a potential conflict of interest.

## Footnotes

^1^Note that the sensor space data (Figure 3) seem to indicate early differences (before 100 ms) between ERFs elicited by distractor and standard in children, however, the first significant differences in the source activity were found at later latency ranges [starting at 165–215 ms, *F*_(1, 14)_ = 6.75, *p*_FDR_ < 0.05]. Thus, we focused our analysis on the given time windows.

^2^The *t*-complex, a group of ERP components elicited at temporal electrodes, is undergoing maturational changes (e.g., Mahajan and McArthur, 2013). These ERP differences might add to age-related distractor effects in the current study and could also account for differences between our EEG and MEG results. The *t*-complex was not in the focus of the current study, yet we investigated our EEG data at temporal electrode sites (T7/T8). We found effects related to distractor processing in both age groups, delayed in children compared to adults. This delay was comparable to our results on the midline (EEG) and the MEG source activation.
